# Effectiveness of an AI- and Gamification-Based Health Literacy Program for Improving Alcohol-Preventive Behaviors Among Hazardous-Drinking Vocational Students: A Quasi-Experimental Study

**DOI:** 10.3390/ijerph23070826

**Published:** 2026-06-23

**Authors:** Potjana Jitjamnong, Chakkrit Ponrachom, Nannapat Ketkosan

**Affiliations:** Faculty of Education, Kasetsart University, Bangkok 10900, Thailand; potjana.j@ku.th (P.J.); nannapat.k@ku.th (N.K.)

**Keywords:** Thailand, health literacy, artificial intelligence, gamification, alcohol-preventive behavior, hazardous drinking, vocational students, quasi-experimental study

## Abstract

**Highlights:**

**Public health relevance—How does this work relate to a public health issue?**

- Hazardous alcohol use among vocational students poses significant health and behavioral risks with long-term social consequences.
- In Thailand, there is a need for tailored health literacy interventions integrating artificial intelligence, online platforms, and gamification to support alcohol preventive behaviors.

**Public health significance—Why is this work of significance to public health?**

- This study develops a health literacy-based intervention integrating artificial intelligence, online platforms, and gamification to promote alcohol-preventive behaviors.
- It strengthens key competencies—accessing, understanding, appraising, and applying health information—for sustainable behavior change.

**Public health implications—What are the key implications or messages for practitioners, policy makers and/or researchers in public health?**

- Alcohol control efforts should move beyond knowledge-based approaches to strengthen health literacy, integrating them into educational and health service systems to achieve effective behavior change.
- Programs integrating artificial intelligence, online platforms, and gamification can serve as scalable models to promote alcohol-preventive behaviors among youth in digital contexts.

**Abstract:**

Low health literacy is associated with risky alcohol use among young people, particularly those exposed to social and environmental factors that normalize drinking. In digital contexts, innovative and engaging interventions are needed to strengthen alcohol-preventive competencies among hazardous drinkers. This study evaluated the effectiveness of an online health literacy promotion program integrating artificial intelligence (AI) and gamification in improving health literacy and alcohol-preventive behaviors among hazardous-drinking vocational students. A quasi-experimental two-group pre-test–post-test design with a 1-month follow-up was conducted among 114 first-year Higher Vocational Certificate students aged 18–20 years in Bangkok, Thailand. Participants were assigned to an intervention group (*n* = 57) or a comparison group (*n* = 57). The intervention group received the ALC Literacy Program, while the comparison group received standard educational materials on alcohol prevention. Data were analyzed using descriptive statistics, chi-square tests, independent t-tests, and two-way mixed-design repeated-measures ANOVA with Bonferroni post hoc comparisons. At baseline, no significant between-group differences were observed. After the intervention and at 1-month follow-up, the intervention group showed significantly greater improvements in both health literacy and alcohol-preventive behaviors than the comparison group (*p* < 0.001). Large interaction effect sizes were observed for health literacy (partial η^2^ = 0.623) and alcohol-preventive behaviors (partial η^2^ = 0.622). These findings indicate that the ALC Literacy Program was effective in enhancing health literacy and strengthening alcohol-preventive behaviors among hazardous-drinking vocational students. This intervention may represent a potentially useful digital health promotion approach for alcohol prevention in educational settings.

## 1. Introduction

Alcohol consumption remains a major public health risk factor with substantial consequences for physical health, mental health, and socioeconomic well-being. The World Health Organization has reported that alcohol is associated with more than 200 diseases and injury conditions and that approximately 2.6 million deaths worldwide in 2019 were attributable to alcohol use. These figures underscore that alcohol use is not merely an individual behavior but a significant public health problem affecting health systems and population quality of life [1].

In Thailand, alcohol consumption continues to be a major public health concern. Among individuals aged 15 years and older, the overall prevalence of alcohol consumption was 28.0%, compared with 31.6% among those aged 20–24 years and 15.0% among those aged 15–19 years [2]. These data indicate that individuals aged 18–20 years, the target group in the present study, are at a critical transitional stage during which the risk of initiating and progressing to hazardous drinking increases. Alcohol initiation also commonly occurs during higher or vocational education, when young people are increasingly exposed to peer influence, social pressure, and greater autonomy in daily life [3].

Students in higher vocational education may be particularly vulnerable to hazardous drinking because social influences can normalize alcohol use, while environmental conditions, especially in urban settings, may increase alcohol exposure [4,5,6]. Although these students may not yet have developed alcohol dependence, hazardous drinking already places them at greater risk of health and social harms and may progress to dependence if it continues [7]. Despite the implementation of preventive and control measures in educational institutions, such as policy measures, screening, brief counseling, and referral systems, these efforts remain insufficiently implemented in practice, particularly for hazardous drinkers [8].

Previous literature suggests that alcohol-preventive behaviors are influenced by both external and internal factors. However, internal factors, such as the ability to avoid risky situations, refusal skills, and self-control, are particularly important for rational decision-making and behavioral regulation [9]. These factors can be strengthened through health literacy, defined as the ability to access, understand, appraise, and apply health information appropriately for health-related decision-making [10]. The health literacy framework proposed by Sørensen et al. was selected as the theoretical foundation of this study because it encompasses four key competencies—access, understanding, appraisal, and application of health information—which are essential for informed decision-making and alcohol-preventive behaviors [10]. Therefore, strengthening health literacy may provide an important mechanism for promoting alcohol-preventive behaviors among hazardous drinkers.

Health literacy may influence alcohol-preventive behaviors through several behavioral mechanisms. Individuals with higher health literacy are more likely to accurately perceive alcohol-related risks, critically evaluate persuasive social influences, and make informed decisions regarding alcohol use [11,12]. In addition, health literacy may strengthen self-regulation and behavioral control by improving individuals’ ability to interpret health information and apply coping strategies in real-life situations [11,13]. Previous studies have shown that limited health literacy is associated with risky alcohol consumption, lower risk perception, and poorer health-related decision-making among adolescents and young adults [11,14,15]. Therefore, strengthening health literacy may not only improve knowledge, but also facilitate behavioral change through cognitive and motivational pathways [12,13].

Recent evidence also suggests that digital and gamified interventions can effectively support addiction prevention and behavior modification among young people [16,17,18]. Gamification mechanisms, including challenges, rewards, feedback, progress tracking, and social interaction, may enhance intrinsic motivation, engagement, and sustained participation in health programs [17,19]. Systematic reviews have reported that gamified digital interventions can improve health behaviors, increase self-management skills, and strengthen adherence to preventive programs, particularly among adolescents and young adults [16,17,18]. In alcohol and substance-use prevention contexts, interactive digital interventions may provide safe environments for practicing refusal skills, self-monitoring, and decision-making in simulated high-risk situations [16,20]. These approaches may therefore support the translation of health literacy competencies into practical alcohol-preventive behaviors.

In the digital era, artificial intelligence, gamification, and online platforms offer considerable potential to support health communication and behavior change. These approaches can be tailored to users’ contexts, enhance intrinsic motivation, and sustain active engagement over time [21,22]. However, empirical evidence in Thailand remains limited regarding interventions that integrate health literacy promotion with AI and gamification to improve alcohol-preventive behaviors among hazardous-drinking vocational students. Therefore, this study aimed to evaluate the effectiveness of an online health literacy promotion program integrating AI and gamification in enhancing health literacy and alcohol-preventive behaviors among hazardous-drinking vocational students in Bangkok, Thailand. We hypothesized that the intervention group would demonstrate significantly greater improvements in health literacy and alcohol-preventive behaviors at post-test and follow-up than the comparison group.

## 2. Materials and Methods

### 2.1. Study Design

This study employed a quasi-experimental two-group pre-test–post-test design with a 1-month follow-up. Assessments were conducted at three time points: baseline, post-intervention, and 1-month follow-up. The intervention group received the ALC Literacy Program, whereas the comparison group received standard educational materials on alcohol prevention during the same study period.

### 2.2. Study Sample

Participants were 114 first-year Higher Vocational Certificate students aged 18–20 years under the Office of the Vocational Education Commission in Bangkok. The age range of 18–20 years was selected because it represents the typical age of first-year Higher Vocational Certificate students in Thailand. This developmental period is characterized by increased autonomy, greater exposure to peer influence, and a higher likelihood of engaging in health-risk behaviors, including alcohol consumption. Previous studies have shown that vocational education students are particularly vulnerable to multiple health-risk behaviors and alcohol-related problems during late adolescence and early adulthood [23,24]. All participants had a history of alcohol consumption and were classified as moderate-risk drinkers based on Alcohol, Smoking and Substance Involvement Screening Test (ASSIST) scores of 11–26. Sample size was estimated using a priori power analysis with a power of 0.80, an effect size of 0.50, and a significance level of 0.05. The minimum required sample size was 102 participants (51 per group). To account for potential attrition, the final sample size was increased by 10%, resulting in 114 participants, with 57 in each group.

The inclusion criteria were as follows: (1) being first-year Higher Vocational Certificate students under the Office of the Vocational Education Commission in Bangkok; (2) being aged 18–20 years; (3) having a history of alcohol consumption and moderate-risk ASSIST scores (11–26); (4) having access to and being able to use a smartphone, computer, or tablet with internet access; (5) being physically able to participate throughout the intervention period; and (6) willingness to complete the questionnaires and participate in the study. Participants were excluded if they were unable to complete the intervention activities or any of the three assessments.

### 2.3. Sampling Procedure and Prevention of Bias

A multistage sampling procedure was used to recruit eligible participants from vocational institutions in Bangkok. First, Bangkok was stratified into three geographical zones: inner, middle, and outer Bangkok. One area from each zone was then selected by simple random sampling. Subsequently, two vocational institutions were selected from the identified areas, with one serving as the intervention site and the other as the comparison site. Eligible students were then screened using the Alcohol, Smoking and Substance Involvement Screening Test (ASSIST) [7], and only those with moderate-risk scores of 11–26 were enrolled. The ASSIST was used only for participant screening and eligibility assessment.

Several measures were used to reduce potential bias. First, participants in the intervention and comparison groups were recruited from different institutions to minimize contamination and co-intervention. Second, baseline characteristics were compared between groups prior to the intervention to assess initial comparability. Third, data collection was conducted during the same period for both groups in order to reduce temporal bias. Finally, all completed questionnaires were reviewed for accuracy and completeness before coding and statistical analysis.

### 2.4. Instruments

The study instruments were developed based on a review of the relevant literature and the health literacy framework, where applicable. Because no standardized instruments were available to comprehensively assess the specific dimensions of health literacy and alcohol-preventive behaviors targeted by the intervention, study-specific questionnaires were developed. Content validity was assessed by a panel of experts, and items with an Index of Item–Objective Congruence (IOC) of 0.50 or higher were retained [25], with IOC values ranging from 0.67 to 1.00. Prior to data collection, the instruments were pilot tested with 40 students from another educational institution in Bangkok who had characteristics similar to those of the target participants. The overall reliability of the instruments was acceptable, with a Cronbach’s alpha coefficient of 0.84.

#### 2.4.1. General Information Questionnaire

The demographic questionnaire was developed based on a literature review. It consisted of nine items covering sex, age, grade point average, current living arrangement, average monthly income, age at first alcohol use, drinking frequency, type of alcoholic beverage consumed, and reasons for first alcohol use.

#### 2.4.2. Health Literacy Questionnaire

The health literacy questionnaire was developed based on the health literacy framework [10]. It consisted of 24 items rated on a 5-point Likert scale: 1 = unable to do, 2 = very difficult, 3 = difficult, 4 = easy, and 5 = very easy. Total scores ranged from 24 to 120. The instrument demonstrated good internal consistency, with a Cronbach’s alpha of 0.83.

#### 2.4.3. Alcohol-Preventive Behaviors Questionnaire

The alcohol-preventive behaviors questionnaire was developed based on a literature review. It comprised 15 items covering three domains: avoidance of situations or opportunities that may lead to alcohol use, refusal of drinking invitations from friends, family members, or close contacts, and self-control in refraining from alcohol use or resisting the urge to drink. Items were rated on a 5-point Likert scale: 0 = not applicable/never encountered the situation, 1 = never, 2 = sometimes, 3 = often, and 4 = always. Total scores ranged from 0 to 60. The instrument demonstrated acceptable internal consistency, with a Cronbach’s alpha of 0.79.

### 2.5. Ethical Considerations

This study was approved by the Human Research Ethics Committee of Boromarajonani College of Nursing, Nopparat Vajira (Approval No. 13/2568; approved 6 October 2025). Written informed consent was obtained from all participants prior to data collection. Participants were informed of their right to refuse or withdraw from the study at any time without consequences. Confidentiality was strictly maintained, and no personally identifiable information was collected. All data were used solely for research purposes.

### 2.6. Developing the Intervention—ALC Literacy Program

The ALC Literacy Program, a health literacy promotion program delivered through an online platform integrating AI and gamification, was developed from a literature review and guided by the health literacy framework. It was designed to strengthen four dimensions of health literacy—access, understanding, appraisal, and application to promote three domains of alcohol-preventive behaviors: avoidance of situations or opportunities that may lead to alcohol use, refusal of drinking invitations from friends, family members, or close contacts, and self-control in refraining from alcohol use or resisting the urge to try alcohol.

The intervention integrated the health literacy framework with AI-assisted learning and gamification mechanisms. AI-supported functions facilitated the processes of accessing, understanding, appraising, and applying alcohol-related health information through interactive information delivery, reflective questioning, personalized educational feedback, and adaptive learning support, thereby promoting active engagement and meaningful learning experiences.

The AI component of the intervention was integrated into a private Facebook group developed specifically for the study to support participants’ access to and evaluation of alcohol-related health information. The intervention incorporated ChatGPT (OpenAI, GPT-3.5) -assisted learning activities to facilitate interactive communication and personalized learning support throughout the program. AI-assisted functions included automated responses, personalized educational feedback, interactive question-and-answer activities, and adaptive learning support based on participants’ responses and learning progress. Participants interacted with the AI system by submitting questions, completing guided learning activities, and receiving immediate feedback during each intervention session. These features were designed to encourage critical evaluation of alcohol-related information, reinforce understanding of alcohol-related risks, and support informed decision-making. Participants accessed the AI-supported activities through smartphones or other digital devices during each session.

**Figure 1:**
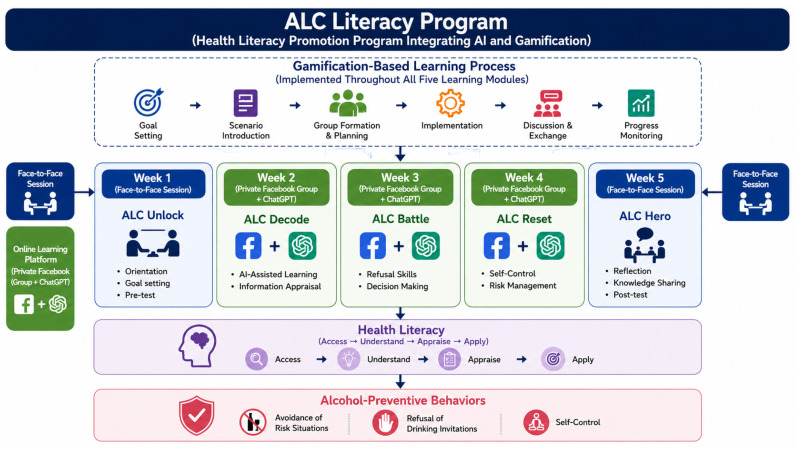
Conceptual Structure of the ALC Literacy Program.

The program supported self-directed learning through a private Facebook group under expert supervision. It followed six gamification stages: (1) defining achievement goals; (2) introducing the scenario and setting goals; (3) group formation and planning; (4) implementation; (5) group discussion and exchange; and (6) summary and progress monitoring. These stages were implemented through the weekly learning activities of the ALC Literacy Program to promote participant engagement, reflection on alcohol-related situations, and ongoing learning throughout the program. The overall conceptual structure and delivery process of the ALC Literacy Program are presented in Figure 1.

Before implementation, the program was reviewed by a panel of experts. The evaluation showed a high level of agreement regarding the appropriateness of the program components and activities (mean = 5.00).

### 2.7. Intervention Description

The ALC Literacy Program was designed to promote progressive learning across five modules, each targeting specific health literacy competencies and alcohol-preventive behavioral skills. The program combined interactive content delivery, AI-assisted inquiry and reflection, peer discussion, scenario-based learning, and gamification elements, including goal setting, task completion, feedback, and progress monitoring.

The intervention group received the ALC Literacy Program for 5 weeks, with one session per week lasting 60–90 min, for a total intervention time of approximately 6 h. The program was delivered through an online platform and could be integrated into student development activities within the participating institutions. The researcher facilitated all sessions and monitored the learning process to ensure adherence to the planned protocol and consistency in program delivery throughout the study period.

For weeks 2–4, the estimated learning duration of 60–90 min per week was based on the time required to review learning materials, complete assigned activities, participate in Facebook-based learning activities, and engage with AI-assisted learning tasks. Participants were allowed to revisit learning materials, Facebook-based activities, and AI-assisted learning tasks multiple times throughout each week according to their individual learning needs.

The program consisted of five sequential learning modules: ALC Unlock, ALC Decode, ALC Battle, ALC Reset, and ALC Hero. These modules corresponded to the weekly intervention sessions delivered from weeks 1 to 5 and were designed to progressively enhance health literacy and promote alcohol-preventive behaviors.

**Figure 2:**
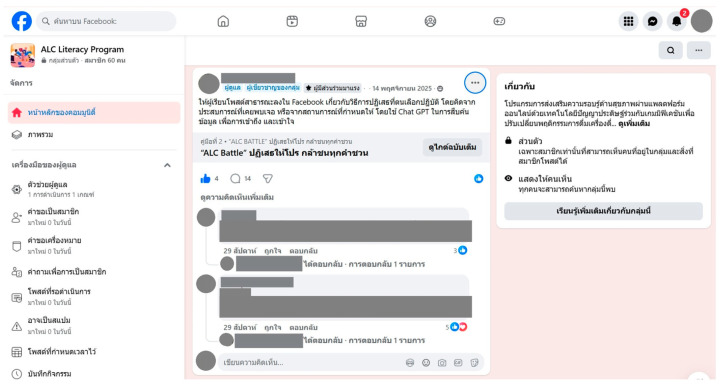
Example of an AI-assisted and gamification-based learning activity (ALC Battle) conducted through the private Facebook group. The Thai-language text shown in the screenshot represents actual participant interactions and instructional content used during the intervention.

In week 1, participants engaged in knowledge-enhancement activities through interactive lectures combined with online quiz-based learning to reinforce motivation. During weeks 2–4, participants engaged in self-directed learning through the Facebook-based platform “ALC Literacy Program.” AI was used as a supportive tool to assist participants in accessing, understanding, appraising, and applying information related to risky situations, refusal strategies, and self-control. An example of an AI-assisted and gamification-based learning activity conducted through the private Facebook group is presented in Figure 2. This process involved critical questioning, evaluation of source credibility, and content synthesis before applying the acquired knowledge to simulated online scenarios. All activities were conducted under the supervision of the researcher and teachers. In week 5, group discussion and reflective activities were conducted to consolidate health literacy development, technology use, and behavioral changes prior to the post-test assessment.

The comparison group did not receive the intervention program. Instead, participants were provided with educational materials on alcohol prevention and completed the same questionnaires during the same assessment periods as the intervention group.

### 2.8. Statistical Analysis

Data were analyzed using IBM SPSS Statistics for Windows, version 22.0. A significance level was set at *p* < 0.05. The intervention (ALC Literacy Program) was treated as the independent variable. The dependent variables included health literacy and alcohol-preventive behaviors.

Descriptive statistics, including frequency, percentage, mean, and standard deviation, were used to describe the general characteristics of the participants. Baseline characteristics between the intervention and comparison groups were compared using the chi-square test for categorical variables and the independent t-test for continuous variables.

Inferential analysis was performed using a two-way mixed-design repeated-measures ANOVA to examine the effects of group (intervention vs. comparison), time (pre-test, post-test, and 1-month follow-up), and the interaction between group and time on the study outcomes. Bonferroni post hoc tests were conducted for pairwise comparisons where appropriate.

Because no statistically significant baseline differences were identified between the intervention and comparison groups, the use of a two-way mixed-design repeated-measures ANOVA was considered appropriate for evaluating changes in outcomes over time as well as differences between groups.

Prior to inferential analysis, statistical assumptions were assessed. Normality was examined using the Shapiro–Wilk test, and homogeneity of variance was evaluated using Levene’s test. The assumption of sphericity was assessed using Mauchly’s test. When sphericity was violated, the degrees of freedom were adjusted using the Greenhouse–Geisser or Huynh–Feldt correction, as appropriate.

## 3. Results

### 3.1. Characteristics of Participants

**Figure 3:**
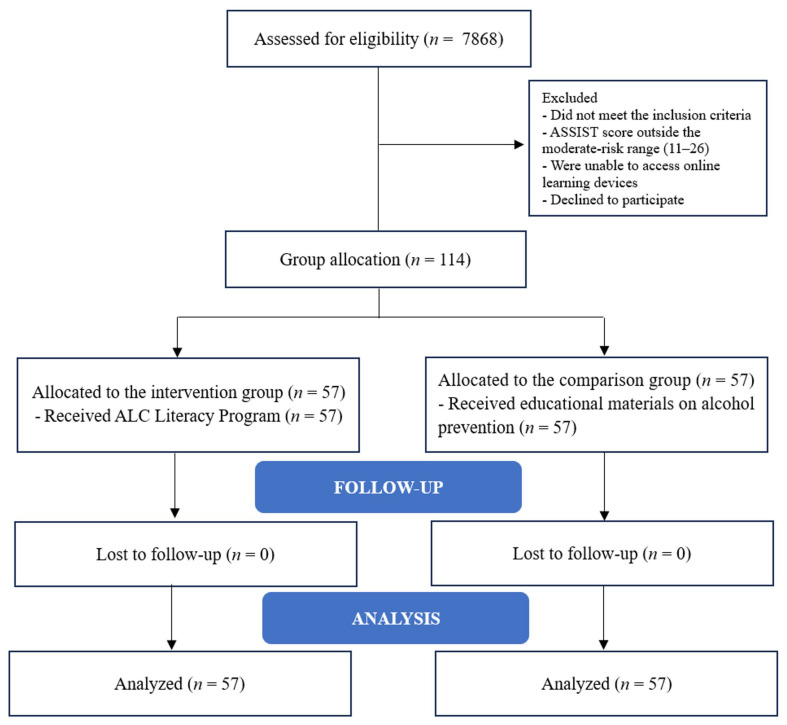
Flow diagram of participant recruitment and follow-up.

Participants were Higher Vocational Certificate students assigned to the intervention group (*n* = 57) or the comparison group (*n* = 57). All participants completed the study and were included in the final analysis, with no loss to follow-up (Figure 3).

No statistically significant between-group differences were found in gender, age, current living arrangement, grade point average, average monthly income, or age at first alcohol use (*p* > 0.05) (Table 1).

At baseline, the most commonly reported reason for first alcohol use in both groups was curiosity or a desire to try alcohol. Monthly drinking was the most frequently reported drinking pattern, and beer was the alcoholic beverage most commonly consumed in both groups. These findings indicate comparable baseline alcohol-related behavioral characteristics between the two groups (Table 2).

### 3.2. Health Literacy and Alcohol-Preventive Behavior Outcomes

**Figure 4:**
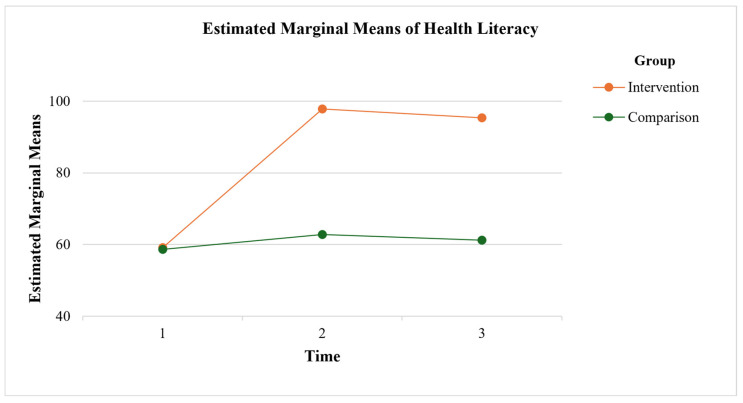
Changes over time in health literacy mean scores between the intervention and comparison groups.

The two-way mixed-design ANOVA revealed significant effects of group, time, and the group × time interaction on health literacy scores (*p* < 0.001). In the intervention group, the mean health literacy score increased from 59.12 (SD = 11.11) at pre-test to 97.84 (SD = 9.11) at post-test and remained high at the 1-month follow-up (95.33, SD = 6.61). In contrast, the comparison group showed only slight changes, with mean scores of 58.63 (SD = 11.42), 62.81 (SD = 12.38), and 61.21 (SD = 10.13) at pre-test, post-test, and the 1-month follow-up, respectively. A large group-by-time interaction effect was observed for health literacy (partial η^2^ = 0.623), indicating that changes over time differed substantially between the intervention and comparison groups (Table 3; Figure 4).

**Figure 5:**
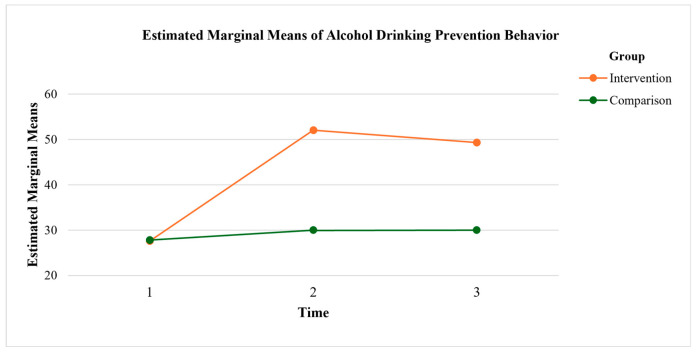
Changes over time in alcohol drinking preventive behavior mean scores between the intervention and comparison groups.

Similarly, significant effects were found for alcohol-preventive behaviors (*p* < 0.001). In the intervention group, the mean score increased from 27.59 (SD = 8.84) at pre-test to 52.07 (SD = 4.98) at post-test and remained high at the 1-month follow-up (49.33, SD = 4.31). In contrast, the comparison group showed only minimal changes, with mean scores of 27.85 (SD = 6.51), 30.00 (SD = 6.43), and 30.02 (SD = 6.01) at pre-test, post-test, and the 1-month follow-up, respectively. Similarly, a large group-by-time interaction effect was observed for alcohol-preventive behaviors (partial η^2^ = 0.622), indicating that the pattern of change over time differed substantially between the intervention and comparison groups (Table 3; Figure 5).

## 4. Discussion

Improving health literacy is an important strategy for promoting alcohol-preventive behaviors and reducing risky alcohol use among young people. In this study, the intervention group showed a significant increase in health literacy after the intervention, and scores remained above baseline at follow-up despite a slight decline over time, whereas no significant change was observed in the comparison group. These findings suggest that the intervention effectively strengthened key health literacy competencies among hazardous drinkers, particularly the abilities to access, understand, appraise, and apply health information for informed decision-making.

These findings are consistent with previous studies showing that well-designed health education interventions can improve health literacy and reduce risky health behaviors. Manthey et al. reported that school-based preventive programs integrating digital media and critical thinking strategies improved alcohol-related health literacy [15]. Similarly, Ponrachom et al. found that interventions based on the health literacy framework enhanced individuals’ abilities to access and interpret health information, contributing to improvements in alcohol-preventive behaviors [26].

The improvement in health literacy observed in this study may be explained by the intervention design, which integrated gamification, AI, and an online learning platform. These approaches may enhance engagement and motivation while providing repeated opportunities to practice accessing, analyzing, and applying health information. Previous studies have shown that gamification elements, such as missions, points, rewards, and feedback, promote active participation and deeper learning [27,28]. AI-assisted learning may also support personalized learning and improve access to relevant health information, thereby strengthening the ability to interpret and apply health knowledge [29].

The improvement in health literacy was accompanied by better alcohol-preventive behaviors in the intervention group. Participants demonstrated significantly higher preventive behavior scores after the intervention, and these remained above baseline at follow-up. This suggests that participants were able to translate the knowledge and skills gained from the program into practical behaviors, including avoiding high-risk drinking situations, refusing drinking invitations, and exercising self-control.

The slight decline in preventive behavior scores at follow-up may reflect the influence of social and environmental factors, such as peer pressure and prevailing social norms related to alcohol use, which are recognized determinants of alcohol consumption among adolescents and young adults [14,30,31]. In addition, the absence of significant behavioral change in the comparison group suggests that behavior change is more likely to occur through structured learning processes that emphasize skill development and active participation rather than information provision alone.

The large effect sizes observed in this study suggest that the intervention may have had a substantial impact on health literacy and alcohol-preventive behaviors. However, these findings should be interpreted with caution, as the magnitude of the observed effects may also have been influenced by contextual factors, including the group-based learning process, the quasi-experimental design, and the use of self-reported measures. Therefore, alternative explanations and potential sources of bias, such as increased participant awareness during the intervention and socially desirable responding, should be considered when interpreting the observed effects.

Overall, these findings highlight the potential of health literacy-based interventions delivered through online platforms integrating AI and gamification as an innovative approach for promoting alcohol-preventive behaviors among hazardous drinkers.

### Strengths and Limitations

This study has several strengths. The quasi-experimental design with intervention and comparison groups allowed a clearer evaluation of program effectiveness. The intervention integrated AI, gamification, and an online learning platform, which enhanced participant engagement and supported the systematic development of health literacy skills. In addition, the follow-up assessment enabled evaluation of the sustainability of program outcomes over time.

Nevertheless, several limitations should be considered when interpreting the findings. First, participants were Higher Vocational Certificate students aged 18–20 years in Bangkok, which may limit the generalizability of the findings to other populations or settings. Second, data were collected using self-reported questionnaires and may therefore be subject to response bias. In addition, the health literacy and alcohol-preventive behavior questionnaires were developed specifically for this study and were not standardized instruments. Although acceptable validity and reliability were established, further validation in different populations is recommended.

In addition, the quasi-experimental design and institutional-level group allocation may have introduced selection bias because participants were not randomly assigned to groups. Although baseline characteristics and outcome measures were comparable between groups prior to the intervention, residual confounding cannot be completely excluded. Therefore, causal inferences should be interpreted with caution. Furthermore, the follow-up period was relatively short and may not fully capture the long-term sustainability of behavioral change or reductions in alcohol-related harm over time. Consequently, caution is warranted when considering the long-term applicability and generalizability of these findings to other populations and contexts.

Future studies employing randomized controlled designs, longer longitudinal follow-up periods (e.g., 6–12 months), and statistical adjustment procedures, such as ANCOVA or multilevel analysis, are recommended to further strengthen causal inference and evaluate the long-term effectiveness of health literacy-based digital interventions on alcohol-preventive behaviors and alcohol-related outcomes.

## 5. Conclusions

This study demonstrated that a health literacy promotion program delivered through an online platform integrating AI and gamification was effective in significantly improving both health literacy and alcohol-preventive behaviors among hazardous-drinking vocational students. Improvements in key health literacy competencies—accessing, understanding, appraising, and applying health information—translated into enhanced abilities to avoid high-risk situations, refuse drinking invitations, and exercise self-control. The findings suggest that structured, skill-based interventions grounded in the health literacy framework may be more effective than information-based approaches alone, while the integration of digital technologies may enhance engagement and support the translation of knowledge into real-life practice. Although a slight decline was observed at follow-up, outcomes remained significantly higher than baseline, indicating short-term sustainability. These findings suggest that the program may have potential as a digital approach for alcohol prevention among youth. However, further studies with longer follow-up periods and randomized designs are needed to confirm its long-term effectiveness and scalability.

## Figures and Tables

**Table 1:** Baseline characteristics of participants in the intervention and comparison groups.

Individual Characteristics	Intervention Group (*n* = 57)		Comparison Group (*n* = 57)		Statistical Analysis
	*n*	%	*n*	%	
**Gender**					
Female	30	52.6	30	52.6	χ^2^ = 0.000 *p* = 1.000
Male	26	45.6	26	45.6	
LGBTQ+	1	1.8	1	1.8	
**Age**					
18 years	22	38.6	24	42.1	χ^2^ = 0.153 *p* = 0.927
19 years	23	40.4	22	38.6	
20 years	12	21.0	11	19.3	
**Current living arrangement**					
Living with parents or a parent	50	87.7	51	89.4	χ^2^ = 1.121 *p* = 0.571
Living with relatives	4	7.0	5	8.8	
Living alone	3	5.3	1	1.8	
**Grade Point Average (GPA)**					
Less than 2.50	10	17.5	9	15.8	χ^2^ = 0.346 *p* = 0.841
2.50–3.00	13	22.9	11	19.3	
Greater than 3.00	34	59.6	37	64.9	
**Average monthly income**					
≤USD 143	31	54.4	29	50.8	χ^2^ = 1.162 *p* = 0.762
USD 143–286	17	29.8	18	31.6	
USD 286–429	8	14.0	7	12.3	
≥USD 429	1	1.8	3	5.3	
**Age at first alcohol use**					
<16 years	49	86.0	46	80.7	χ^2^ = 0.586 *p* = 0.451
≥16 years	8	14.0	11	19.3	

**Table 2:** Baseline alcohol-related characteristics of participants in the intervention and comparison groups.

Individual Characteristics	Intervention Group (*n* = 57)		Comparison Group (*n* = 57)	
	*n*	%	*n*	%
**Reason for first alcohol consumption** (multiple responses allowed)				
Curiosity/personal desire to try	48	84.2	45	78.9
Peer invitation	14	24.6	16	28.1
Family member invitation	13	22.8	15	26.3
Social media/influencer influence	7	12.3	5	8.8
Peer imitation	4	7.0	2	3.5
Being challenged	3	5.3	2	3.5
Being forced	1	1.8	0	0.0
**Frequency of alcohol consumption**				
Daily (7 days/week)	1	1.8	0	0.0
Almost daily (5–6 days/week)	1	1.8	0	0.0
Every other day (3–4 days/week)	4	7.0	2	3.5
Weekly (1–2 days/week or 4–8 days/month)	13	22.8	15	26.3
Monthly (1–3 days/month)	38	66.6	40	70.2
**Type of alcoholic beverage most frequently consumed**				
Beer	37	65.0	36	63.2
Wine/Champagne/Fruit wine	2	3.5	2	3.5
Colored spirits (e.g., Chiang Chun)	2	3.5	0	0.0
Mixed alcoholic drinks (e.g., alcoholic smoothies, bucket drinks, cocktails)	6	10.5	10	17.5
Wine coolers/fruit-flavored alcoholic beverages	4	7.0	4	7.0
Distilled spirits (e.g., gin, rum, whisky, brandy)	6	10.5	5	8.8

**Table 3:** Health literacy and alcohol drinking preventive behavior mean scores and two way mixed-design analysis of variance results.

Scales and Measurements	Intervention Group (*n* = 57)	Comparison Group (*n* = 57)		Group	Time	Group*Time Interaction
	Mean (SD)	Mean (SD)				
**Health literacy**						
Pre-test	59.12 (11.11) ^A,a^	58.63 (11.42) ^A,a^	F	231.598	267.673	185.157
Post-test	97.84 (9.11) ^A,b^	62.81 (12.38) ^B,a^	*p*	<0.001	<0.001	<0.001
Follow-up (1 month)	95.33 (6.61) ^A,b^	61.21 (10.13) ^B,a^	η^2^	0.674	0.705	0.623
**Alcohol drinking prevention behavior**						
Pre-test	27.59 (8.84) ^A,a^	27.85 (6.51) ^A,a^	F	214.646	266.279	184.126
Post-test	52.07 (4.98) ^A,b^	30.00 (6.43) ^B,a^	*p*	<0.001	<0.001	<0.001
Follow-up (1 month)	49.33 (4.31) ^A,b^	30.02 (6.01) ^B,a^	η^2^	0.657	0.704	0.622

F = two-way mixed-design ANOVA; η^2^ = partial eta squared. Different uppercase letters in the same row indicate significant differences between intervention and comparison groups. Different lowercase letters in the same column indicate significant differences between measurement times.

## Data Availability

The current study’s data are not publicly available due to ethical considerations and the protection of the participant’s confidentiality.

## Abbreviations

The following abbreviations are used in this manuscript:

AI	Artificial Intelligence
ALC	Alcohol
ASSIST	Alcohol, Smoking and Substance Involvement Screening Test
